# MicroRNA-195 prevents dendritic degeneration and neuron death in rats following chronic brain hypoperfusion

**DOI:** 10.1038/cddis.2017.243

**Published:** 2017-06-01

**Authors:** Xin Chen, Xue-Mei Jiang, Lin-Jing Zhao, Lin-Lin Sun, Mei-Ling Yan, You Tian, Shuai Zhang, Ming-Jing Duan, Hong-Mei Zhao, Wen-Rui Li, Yang-Yang Hao, Li-Bo Wang, Qiao-Jie Xiong, Jing Ai

**Affiliations:** 1 Department of Pharmacology, The State-Province Key Laboratories of Biomedicine-Pharmaceutics of China, College of Pharmacy of Harbin Medical University, Harbin 150086, China; 2 Department of Neurosurgery, The First Affiliated Hospital of Harbin Medical University, Harbin 150001, China; 3 Department of Medicinal Chemistry and Natural Medicine Chemistry, College of Pharmacy of Harbin Medical University, Harbin 150086, China; 4 Department of Neurobiology and Behavior, SUNY at Stony Brook, Stony Brook, NY 1794, USA

## Abstract

**Impaired synaptic plasticity and neuron loss are hallmarks of Alzheimer’s disease and vascular dementia. Here, we found that chronic brain hypoperfusion (CBH) by bilateral common carotid artery occlusion (2VO) decreased the total length, numbers and crossings of dendrites and caused neuron death in rat hippocampi and cortices. It also led to increase in N-terminal** ***β*****-amyloid precursor protein (N-APP) and death receptor-6 (DR6) protein levels and in the activation of caspase-3 and caspase-6. Further study showed that DR6 protein was downregulated by** ***miR-195*** **overexpression, upregulated by** ***miR-195*** **inhibition, and unchanged by binding-site mutation and miR-masks. Knockdown of endogenous** ***miR-195*** **by lentiviral vector-mediated overexpression of its antisense molecule (lenti-pre-AMO-*****miR-195*****) decreased the total length, numbers and crossings of dendrites and neuron death, upregulated N-APP and DR6 levels, and elevated cleaved caspase-3 and caspase-6 levels. Overexpression of** ***miR-195*** **using lenti-pre-*****miR-195*** **prevented these changes triggered by 2VO. We conclude that** ***miR-195*** **is involved in CBH-induced dendritic degeneration and neuron death through activation of the N-APP/DR6/caspase pathway.**

Brain atrophy induced by neuronal loss is a common hallmark of Alzheimer’s disease (AD) and vascular dementia (VaD), whose other hallmarks include A*β* plaques, tau hyperphosphorylation and cholinergic neuron dysfunction.^1, 2, 3^ Neuronal cell death, a main cause of neuronal loss, can be triggered from either the cell body (soma) or the neurites, and the two processes are controlled by different molecular events.^4, 5, 6, 7^ Importantly, impaired neurite outgrowth and A*β* toxicity-induced abnormalities in synaptic function and axonal connectivity long precede somatic cell death in hippocampal neurons,^4^ suggesting that inhibition of neurite degeneration would be a potential target to prevent or treat AD and VaD. The morphology of dendrites, one type of neurite, has a profound impact on neuronal information processing because the dendritic branching pattern determines the efficacy with which synaptic information is transmitted to the soma and dynamically integrates synaptic input.^8, 9^ Previous studies reported that impaired dendritic ramification was found in both Tg2576 mice and sporadic AD rats.^10, 11^ Although chronic brain hypoperfusion (CBH), which is a preclinical condition of mild cognitive impairment (MCI) and may precede AD and VaD,^12, 13, 14^ can produce neuron death,^15, 16, 17^ whether CBH can induce dendritic morphological remodeling is currently unclear.

It has been known that neurotrophic factors, including nerve growth factor (NGF), brain-derived neurotrophic factor, neurotrophin-3 (NT-3) and NT-4, regulate cell fate decisions, axon growth and dendrite pruning through the Trk family of tyrosine kinase receptors and p75NTR.^18^ Interest, Nikolaev *et al.*^5^ reported that NGF deprivation could trigger the timely axonal pruning and neuron death mediated by caspase-6 and caspase-3 respectively, which was dependent on activated death receptor-6 (DR6, also known as TNFRSF21), an orphan tumor necrosis factor receptor superfamily member. Further study demonstrated that this process could be triggered by N-terminal *β*-amyloid precursor protein (N-APP) generated by trophic factor deprivation-induced cleavage of APP by *β*-site APP cleaving enzyme 1 (BACE1) because N-APP acts as a necessary and sufficient ligand for DR6.^5^ More importantly, recent research showed that Bcl-xL-induced impairment of neurite outgrowth was dependent on upregulation of DR6 (ref. 6) and that DR6 expression was upregulated within dystrophic neurites in and around amyloid plaques in adult Down’s syndrome (DS) patients^19^ and after hypoxia.^6^ Whether DR6 participates in CBH-induced remodeling of dendritic morphology and neuronal loss is of interest.

Animal studies demonstrated that CBH could induce impairment of cognition accompanied by upregulation of APP and BACE1, and hyperphosphorylation of tau as well as hippocampal neuronal and synaptic loss.^1, 20, 21, 22, 23, 24^ In a series of previous studies, we demonstrated that CBH induced by bilateral common carotid artery occlusion (2VO) impaired the learning and memory ability of rats with A*β* aggregation and tau hyperphosphorylation, which were found to be generated by upregulation of both APP and BACE1 mediated by low expression of *microRNA-195* (*miR-195*).^23, 24^ These results led us to speculate that *miR-195* may regulate dendritic morphology and neuron loss by increasing the production of N-APP, which binds with DR6 following CBH.

## Results

### CBH promotes dendrite degeneration and neuron loss in rat hippocampi and cortices

**Figure 1:**
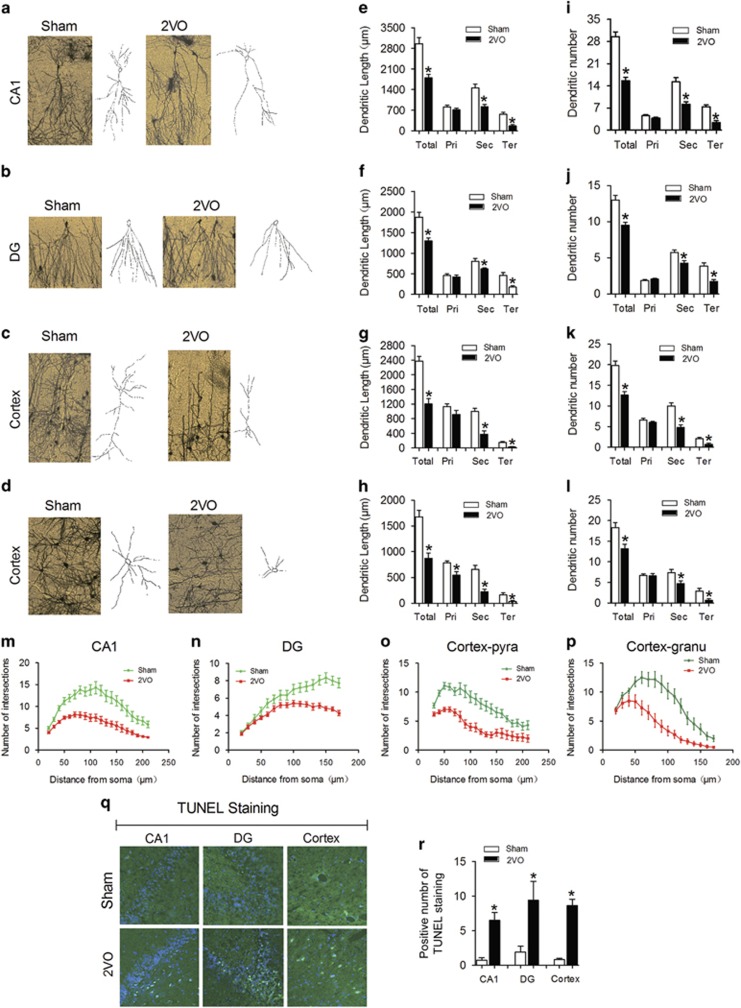
Dendritic complexity deficits and neuron loss in hippocampi and cortices of 2VO rats. (**a**–**d**) Representative photomicrographs (left) and tracing images (right) of dendritic arborization in hippocampal CA1 pyramidal neurons (**a**), DG granular neurons (**b**), cortical pyramidal neurons (**c**) and cortical granular neurons (**d**) from sham and 2VO rats. (**e**–**h**) Quantification of the length of dendrites in hippocampal CA1 pyramidal neurons (**e**), DG granular neurons (**f**), cortical pyramidal neurons (**g**) and cortical granular neurons (**h**). (**i**–**l**) Quantification of the number of dendrites in hippocampal CA1 pyramidal neurons (**i**), DG granular neurons (**j**), cortical pyramidal neurons (**k**) and cortical granular neurons (**l**). (**m**–**p**) Sholl analysis of the number of intersections of dendrites in hippocampal CA1 pyramidal neurons (**m**), DG granular neurons (**n**), cortical pyramidal neurons (**o**) and cortical granular neurons (**p**). (Data are reported as the mean±S.E.M., *n*=15. *P<0.05 *versus* sham. Pri, primary dendrite; Sec, secondary dendrite; Ter, tertiary dendrite; Total, Total dendrite). (**q**) Representative TUNEL photomicrographs in hippocampal CA1 and DG and cortex of sham and 2VO rats at × 20 magnification. (**r**) Statistical analysis of TUNEL-positive cells in hippocampal CA1 and DG and cortex of sham and 2VO rats. Mean±S.E.M., *n*=5, *P<0.05 *versus* sham

To explore whether CBH could induce dendrite degeneration and neuron loss, permanent bilateral occlusion of the common carotid arteries (2VO) was surgically performed in rats as described in previous reports.^23, 24, 25^ We first observed the morphology of dendrites in the CA1 and dentate gyrus (DG) regions of the hippocampus and in the cortex by Golgi staining.^11^ We found significant decreases in the total length and numbers of dendrites of pyramidal and granular neurons in the hippocampal CA1 (Figures 1a, e and i) and DG regions (Figures 1b, f and j) in 2VO rats compared with those of the sham control group. Interestingly, although the length and numbers of secondary and tertiary dendrites were reduced in both pyramidal and granular neurons in the hippocampal CA1 and DG regions of 2VO rats, those parameters were not changed in primary dendrites (Figures 1e, f, i and j). Similar results were observed in cortical pyramidal neurons (Figures 1c, g and k) and granular neurons (Figures 1d, h and l), and the primary dendrites of cortical granular neurons in 2VO rats were shorter than those in sham control rats (Figure 1h). In addition, the dendritic crossings as assessed by Sholl analysis were significantly reduced in all the hippocampal CA1 pyramidal (Figure 1m, *F*_(1, 28)_=32, *P*<0.0001) and DG granular neurons (Figure 1n, *F*_(1, 28)_=10.41, *P*=0.0032) as well as in cortical pyramidal (Figure 1o, *F*_(1, 28)_=4.01, *P*=0.049) and granular neurons (Figure 1p, *F*_(1, 28)_=12.23, *P*=0.0014) in 2VO rats compared with sham controls.

It has been reported that oxygen-glucose deprivation in brain slices and bilateral carotid artery stenosis in mice can induce neuron apoptosis.^15, 17^ Hence, we evaluated whether CBH in rats could elicit neuron death. By performing TdT-mediated UTP nick end labeling (TUNEL), we observed significantly increased neuron death in all cortices and hippocampal CA1 and DG regions (Figures 1q and r). These results conclusively demonstrated the ability of CBH to promote dendritic degeneration and neuronal death in rat hippocampi and cortices.

### CBH activates the N-APP/DR6 pathway in rat hippocampi and cortices

**Figure 2:**
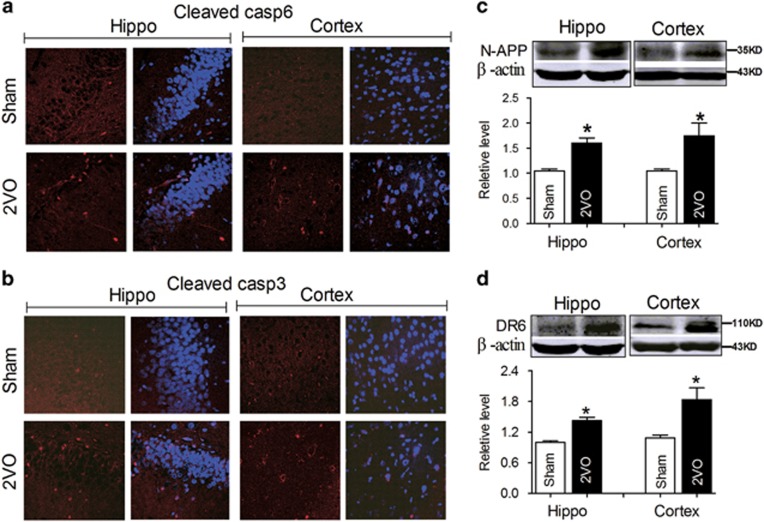
2VO activates the N-APP/DR6/caspase pathways in rats. (**a**) Activated caspase-6 (red) in the hippocampi and cortices of 2VO rats shown by immunofluorescence staining. (**b**) Activated caspase-3 (red) in the hippocampi and cortices of 2VO rats shown by immunofluorescence staining (nuclei are labeled with blue DAPI, and the magnification is × 20). (**c** and **d**) CBH upregulated N-APP and DR6 expression in rat hippocampi and cortices. Mean±S.E.M., *n*=6, *P<0.05 *versus* sham

Previous research demonstrated that increases in N-APP triggered axonal degeneration and neuronal cell body apoptosis via binding with DR6 depended on caspase-6 and caspase-3 respectively.^5, 26^ In addition, the apoptotic process induced by CBH was associated with caspase-3.^15^ We therefore evaluated the activation of caspase-6 and caspase-3 using immunofluorescence staining. As predicted, the positive immunofluorescence signal of the cleaved caspase-6 and caspase-3 in the hippocampi and cortices of 2VO rats were higher than in sham control rats (Figures 2a and b), consistent with a previous report.^15^ Our previous studies demonstrated that 2VO significantly impaired the learning and memory ability of rats and caused A*β* aggregation and tau hyperphosphorylation by upregulating the expression of APP and BACE1 protein,^23, 24^ which inevitably results in the increase of N-APP. More importantly, DR6 expression was reported to be increased in the hippocampus after hypoxia.^6^ We hence hypothesized that the activation of caspase-6 and caspase-3 in the hippocampus and cortex might be associated with elevated N-APP and DR6 in CBH rats. By western blotting, we found that both N-APP and DR6 protein levels were higher in the hippocampi and cortices of 2VO rats than in those of sham control rats (Figures 2c and d). These data suggested that dendrite degeneration and neuron death in rat hippocampi and cortices following CBH involved the activated N-APP/DR6/caspase pathway.

### Knockdown of *miR-195* elicits dendrite degeneration and neuron loss both *in vivo* and *in vitro*

**Figure 3:**
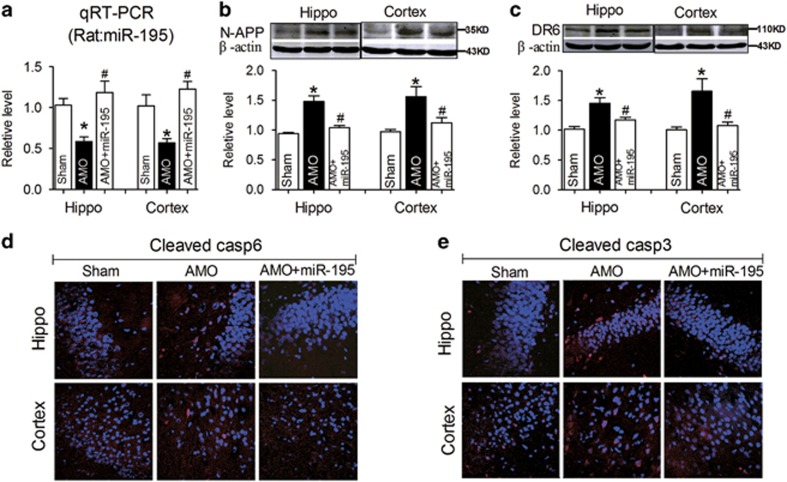
Knockdown of *miR-195* activates the N-APP/DR6/caspase pathway in rats. (**a**) *miR-195* expression in the hippocampi and cortices of rats at the 8th week following stereotaxic injection of lenti-pre-AMO-*miR-195* and/or lenti-pre-*miR-195* was detected by qRT-PCR. (**b** and **c**) Lenti-pre-AMO-*miR-195* upregulated the expression of N-APP and DR6 in rat hippocampi and cortices, mean±S.E.M., *n*=6, *P<0.05 *versus* sham; ^#^*P*<0.05 *versus* lenti-pre-AMO-*miR-195*. (**d** and **e**) Lenti-pre-AMO-*miR-195* induced the activation of caspase-6 and caspase-3 in rat hippocampi and cortices. Nuclei were labeled with blue DAPI, and the magnification was × 20

We next decided to explore the molecular mechanism. Our previous study demonstrated that CBH-induced A*β* deposition in rat hippocampi and temporal lobe cortices was due to upregulation of the APP and BACE1 proteins, and these changes were regulated by the reduction of *miR-195* expression.^23^ We speculated that *miR-195* might also be involved in the process. To clarify this issue, lentiviral vectors (2.0 *μ*l) containing anti-*miR-195* oligonucleotide fragments (lenti-pre-AMO-*miR-195*) and *miR-195* oligonucleotide fragments (lenti-pre-*miR-195*) were developed and stereotaxically injected into the bilateral hippocampal CA1 subfields of normal rats to assess the role of *miR-195* in dendrite degeneration and neuronal death. Consistent with our previous study,^23, 24^ the application of lenti-pre-AMO-*miR-195* inhibited *miR-195* expression in rat hippocampi and cortices at the 8th week compared with that in rats injected with negative control (NC), while co-injection of lenti-pre-*miR-195* reversed the reduction of *miR-195* expression (Figure 3a). As predicted, lenti-pre-AMO-*miR-195* injection successfully elicited the upregulation of N-APP and DR6, which was prevented by co-injection of lenti-pre-*miR-195* (Figures 3b and c). In addition, similar effects of *miR-195* were observed in the changes in cleaved caspase-6 (Figure 3d) and caspase-3 (Figure 3e) by immunofluorescence staining.

**Figure 4:**
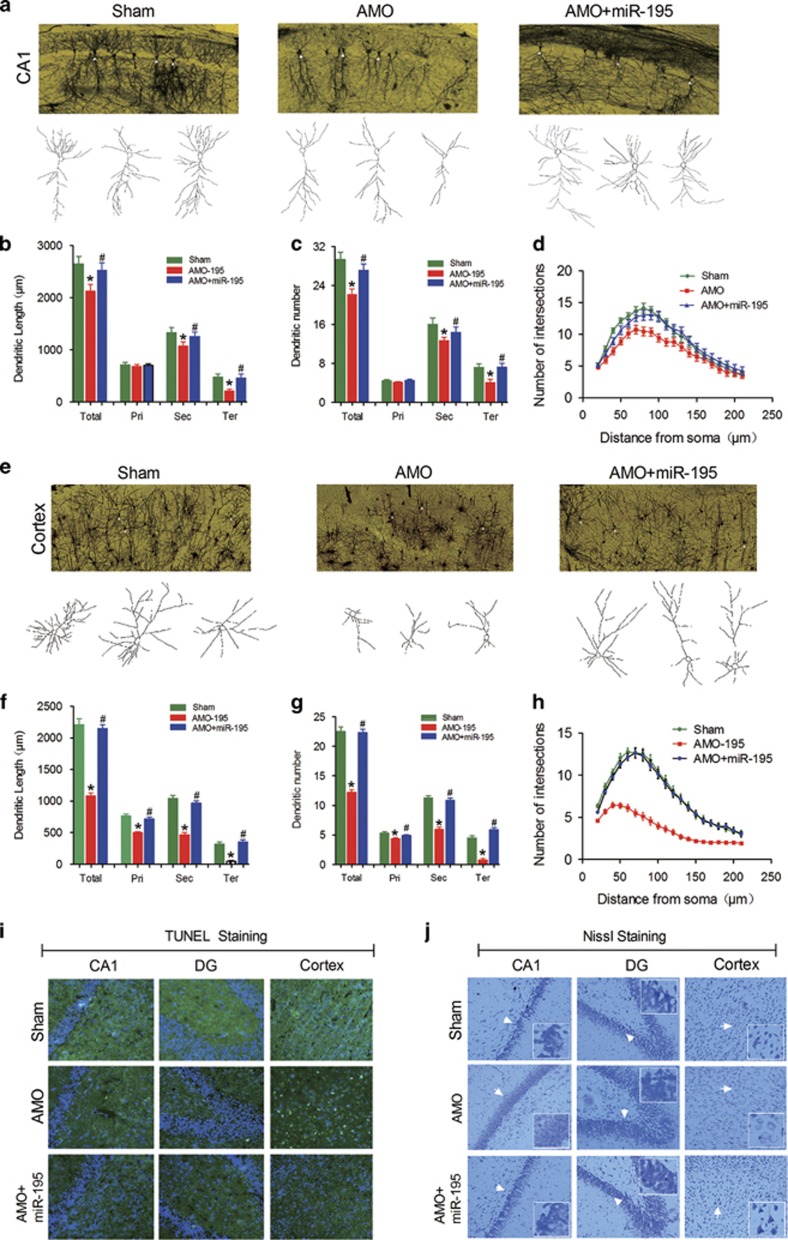
Knockdown of *miR-195* induces dendritic complexity deficits and neuron loss in rats. (**a**) Typical photomicrographs (top) and tracing images (bottom) of dendritic arborization in hippocampal CA1 pyramidal neurons from rats treated with lenti-pre-AMO-*miR-195*, lenti-pre-AMO-*miR-195*+lenti-pre-*miR-195* and NC. (**b**–**d**) Quantification of the length, the number and the intersection number of dendrites in hippocampal CA1 pyramidal neurons from rats treated with lenti-pre-AMO-*miR-195*, lenti-pre-AMO-*miR-195*+lenti-pre-*miR-195*, and NC. (**e**) Typical photomicrographs (top) and tracing images (bottom) of dendritic arborization in cortical pyramidal neurons from rats treated with lenti-pre-AMO-*miR-195*, lenti-pre-AMO-*miR-195*+lenti-pre-*miR-195* and NC. (**f**–**h**) Quantification of the length, the number and the intersection number of dendrites in cortical pyramidal neurons from rats treated with lenti-pre-AMO-*miR-195*, lenti-pre-AMO-*miR-195*+lenti-pre-*miR-195* and NC. (Mean±S.E.M., *n*=20 in hippocampi, *n*=30 in cortices. *P<0.05 *versus* sham; ^#^*P*<0.05 *versus* lenti-pre-AMO-*miR-195*. Pri, primary dendrite; Sec, secondary dendrite; Ter, tertiary dendrite; Total, Total dendrite). (**i**) TUNEL staining revealed that *miR-195* knockdown triggers neuron loss, *n*=3. The magnification was × 20. (**j**) Representative photomicrographs of Nissl staining in hippocampal CA1, DG and cortex from rats treated with lenti-pre-AMO-*miR-195*, lenti-pre-AMO-*miR-195*+lenti-pre-*miR-195* and NC at × 20 magnification, *n*=3

Accordingly, Golgi staining data showed that, as seen in 2VO rats, lenti-pre-AMO-*miR-195* application resulted markedly shortened the dendritic length and lessened the dendritic numbers in secondary and tertiary dendrites, but not in primary dendrites, of pyramidal neurons in the CA1 region (Figures 4a–c), with significantly reduced dendritic crossings by Sholl analysis compared with the NC injection group (Figure 4d, *F*_(2, 57)_=6.94, *P*<0.002). Interestingly, it failed to elicit any changes in granular neurons in the DG region (Supplementary Figures S1a–d). Importantly, lenti-pre-AMO-*miR-195* injection also significantly reduced the dendritic length and numbers as well as the dendritic crossings of pyramidal (Figures 4e–h; for h: *F*_(2, 87)_=82.32, *P*<0.0001) and granular neurons (Supplementary Figures S1e–h) in the cortex relative to NC treated rats. Co-injection of lenti-pre-*miR-195* successfully prevented all the changes induced by lenti-pre-AMO-*miR-195* injection (Figures 4a–h).

Thereafter, both TUNEL and Nissl staining techniques were performed to observe whether knockdown of *miR-195* could induce neuron death. We found that knockdown of *miR-195* by lenti-pre-AMO-*miR-195* injection elicited significantly increased positive TUNEL signals in the hippocampal CA1 area and cortex (Figure 4i) and caused decreased Nissl staining signals compared with sham control rats (Figure 4j); both effects were prevented by co-injection with lenti-pre-*miR-195* (Figures 4i and j). In contrast, either lenti-pre-AMO-*miR-195* injection alone or co-injection of lenti-pre-AMO-*miR-195* with lenti-pre- *miR-195* failed to influence neuron death and Nissl staining signal in the DG region of hippocampi compared with sham control rats (Figures 4i and j). The phenomenon was similar to the effect of lenti-pre-AMO-*miR-195* on dendrite degeneration evaluated by Golgi staining (Supplementary Figures S1a–d).

**Figure 5:**
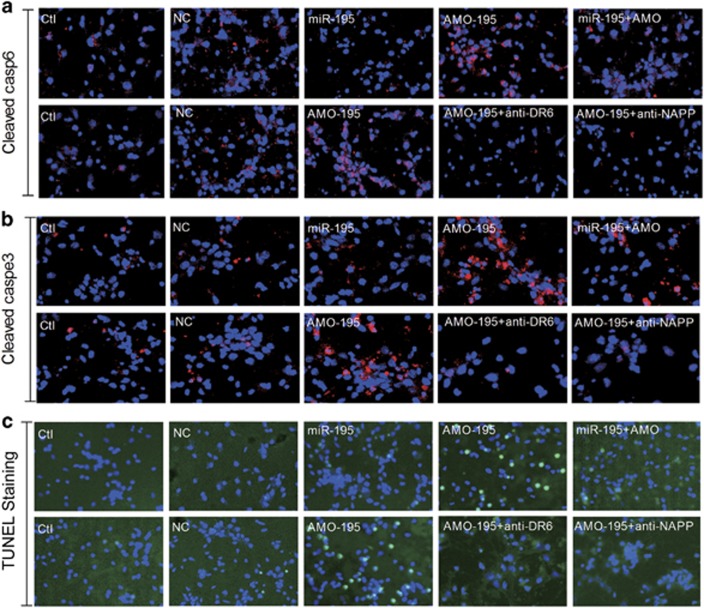
Downregulation of *miR-195* activates caspase and neuron death in a manner dependent on the DR6/N-APP pathway. (**a** and **b**) Representative confocal images of cleaved caspase-6 and cleaved caspase-3 positive signal in NRNs treated with NC, *miR-195*, AMO-195 or AMO-195+antibodies (anti-DR6 or anti-N-APP) at × 20 magnification. (**c**) Representative TUNEL images in NRNs treated with NC, *miR-195*, AMO-195 or AMO-195+antibodies (anti-DR6 or anti-N-APP) at × 20 magnification, *n*=3

To further demonstrate the hypothesis that knockdown of *miR-195* induced dendrite degeneration and neuron loss in a manner dependent on the N-APP/DR6/caspase pathway, we used an *in vitro* strategy. We first transfected *miR-195* and AMO-195 into primary cultured neonatal rat neurons (NRNs) using X-treme GENE siRNA transfection reagent. The transfection efficiency of *miR-195* and AMO-195 into NRNs was verified using qRT-PCR (Supplementary Figure S2). We found that *miR-195* mimics significantly inhibited, while AMO-195 markedly increased, the levels of cleaved caspase-6 and caspase-3, and these changes did not occur upon co-transfection of *miR-195* mimics and AMO-195 (Figures 5a and b). Interestingly, upon co-transfection of AMO-195 with either anti-DR6 antibody or anti-NAPP antibody, the increase of cleaved caspase-6 and caspase-3 induced by AMO-195 alone was blocked (Figures 5a and b). Consistent with these results, either anti-DR6 antibody or anti-NAPP antibody could block AMO-195-induced neuron death (Figure 5c). These results soundly demonstrated that knockdown of *miR-195* could induce dendrite degeneration and neuron loss via the activation of the N-APP/DR6/caspase pathway.

**Figure 6:**
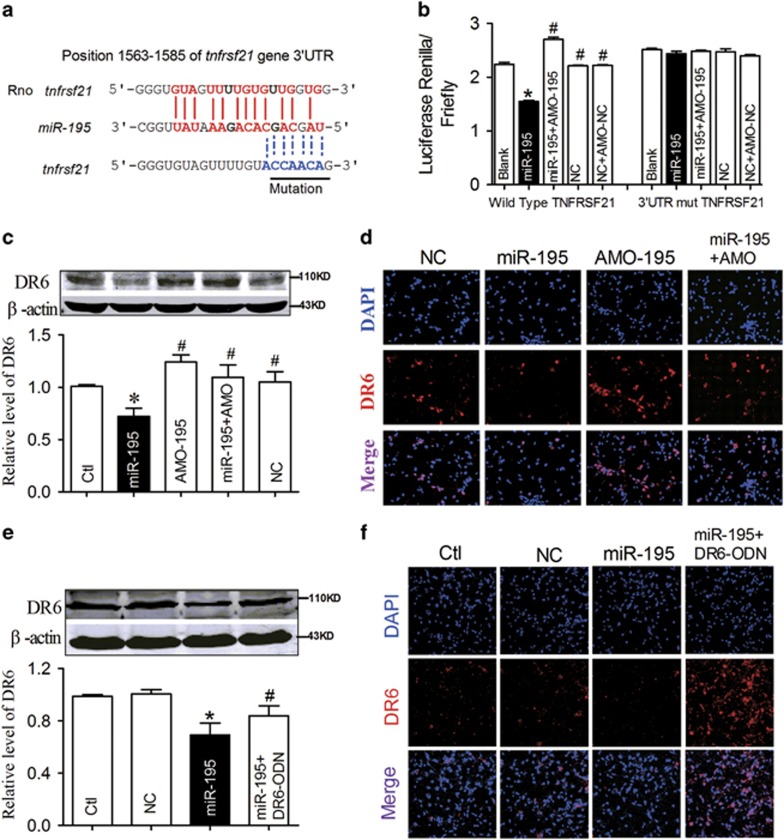
DR6 is a direct target of *miR-195*. (**a**) Schematic of the predicted *miR-195* binding site in the DR6 3′UTR in rats. Nucleotides with Watson–Crick complementarity are connected by ‘|’. The mutations made to the genes are underlined. (**b**) A luciferase reporter gene assay detected the direct interaction between *miR-195* and its binding sites in the 3′UTR of the DR6 mRNA in HEK293T cells. The relative luciferase activity was determined after co-transfection with DR6 3′-UTR or Mut-DR6 3′-UTR plasmids and *miR-195*, *miR-195*+AMO-*miR-195* or NC (*P<0.05 *versus* blank, ^#^*P*<0.05 *versus miR-195*). (**c-d**) The effects of *miR-195* on endogenous DR6 expression in NRNs by western blotting and immunofluorescence staining after the neurons were transfected with *miR-195*, AMO-195, *miR-195*+AMO-195 or NC. (**e** and **f**) Derepression of DR6 by DR6-ODN in NRNs, determined by western blotting and immunofluorescence staining. (*n*=6, *P<0.05 *versus* control, ^#^*P*<0.05 *versus miR-195*. Representative photomicrographs are at a magnification of × 20.)

The next issue is that knockdown of *miR-195* induced the elevation of N-APP because of its upregulating action on APP and BACE1;^23^ how, then, does *miR-195* affect the expression of DR6? By searching the microRNA database RNAhybrid, we found that there was a *miR-195* binding site within the *tnfrsf21* gene, which encodes the DR6 protein, at 1563–1585 bp in the 3′UTR (Figure 6a). The full length of the 3′UTR of *tnfrsf21* containing the *miR-195* binding sites was then cloned into a luciferase-expressing reporter plasmid, and we assessed the effects of *miR-195* on reporter activities in HEK293T cells. As illustrated in Figure 6b, co-transfection of *miR-195* with the plasmid consistently decreased the luciferase activity relative to that for transfection of the plasmid alone, whereas application of AMO-195 or mutation of the binding sites abolished the silencing effect of *miR-195* (Figure 6b; for wild type: *F*_(4, 14)_=258.3, *P*<0.0001; for mutant: *F*_(4, 14)_=1.447, *P*=0.2887). We then evaluated whether *miR-195* could affect DR6 protein expression. *MiR-195* and AMO-195 were transfected into NRNs using X-treme GENE siRNA transfection reagent. Western blot analysis showed that overexpression of *miR-195* significantly inhibited DR6 protein level, while co-transfection of AMO-195 significantly inhibited its effect on DR6 expression (Figure 6c). The phenomenon was further demonstrated by immunofluorescence staining (Figure 6d).

To further clarify the direct effect of *miR-195* on DR6 expression, a miRNA-masking antisense oligodeoxynucleotides (ODN) for the *tnfrsf21* gene was designed and synthesized. The ODN was fully complementary to the predicted *miR-195* binding sites in the 3′UTRs of the *tnfrsf21* gene and was labeled as DR6-ODN. Co-transfection of DR6-ODN with *miR-195* into NRNs blocked the inhibitory effects of *miR-195* on DR6 (Figure 6e), as was further indicated by the prevention of DR6 immunofluorescence signal reduction induced by *miR-195* transfection (Figure 6f). These results indicated that *miR-195* knockdown-induced dendrite degeneration and neuron loss were not only due to its action on overproduction of N-APP but also correlated with its direct action on the upregulation of DR6 expression.

### Gain of *miR-195* reverses the dendrite degeneration and neuron loss in hippocampal CA1 and cortex induced by 2VO

**Figure 7:**
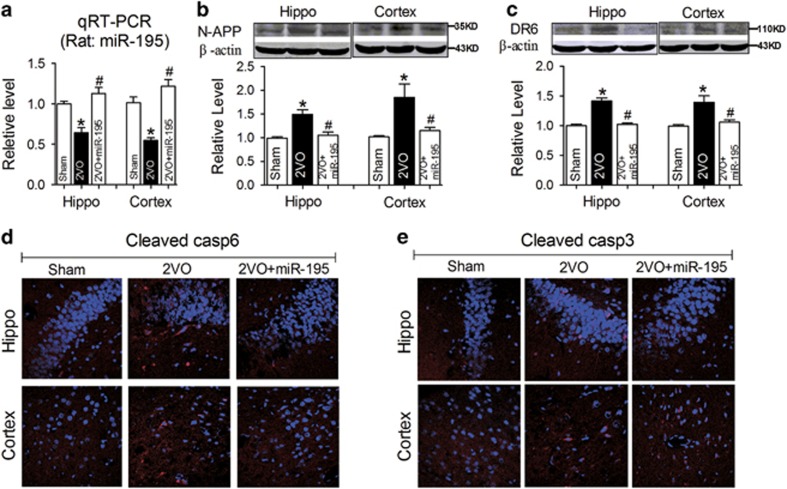
Overexpression of *miR-195* prevents 2VO-induced activation of the N-APP/DR6/caspase pathway. (**a**) *miR-195* levels in the hippocampi and cortices of 2VO rats with or without lenti-pre-*miR-195* treatment. (**b** and **c**) Lenti-pre-*miR-195* prevented the increase of N-APP and DR6 expression in the hippocampi and cortices of 2VO rats. Mean±S.E.M., *n*=6, *P<0.05 *versus* sham, ^#^*P*<0.05 *versus* 2VO. (**d** and **e**) Lenti-pre-*miR-195* inhibited the cleavage of caspase-6 and caspase-3 in the hippocampus and cortex of 2VO rats. The magnification was × 20

The next issue was whether *miR-195* indeed plays a significant role in 2VO-induced dendrite degeneration and neuron loss. To address this query, lenti-pre-*miR-195* was injected into the hippocampal CA1 region of 2VO rats. As in our previous study, the *miR-195* level was significantly increased in the hippocampi and cortices of 2VO rats when lenti-pre-*miR-195* was injected (Figure 7a), suggesting the successful gain of function of *miR-195* in 2VO rats. As predicted, the increases in N-APP and DR6 expression in both the hippocampi and the cortices of 2VO rats were prevented by lenti-pre-*miR-195* injection (Figures 7b and c). Accordingly, by immunofluorescence staining, the increases of cleaved caspase-6 and caspase-3 in both the hippocampi and the cortices of 2VO rats were also markedly inhibited by lenti-pre-*miR-195* application (Figures 7d and e).

**Figure 8:**
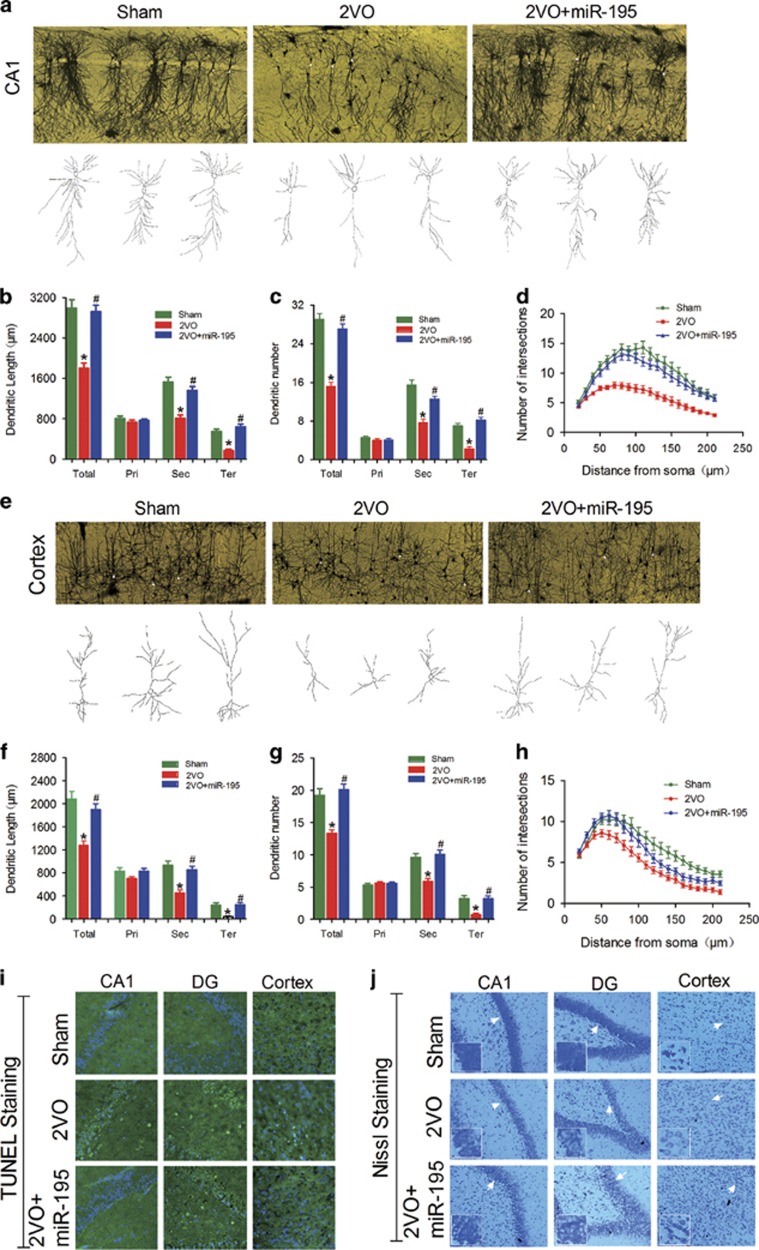
*miR-195* attenuates dendritic complexity deficits and neuron loss in 2VO rats. (**a**) Typical photomicrographs (top) and tracing images (bottom) of dendritic arborization in hippocampal CA1 pyramidal neurons of 2VO rats with or without lenti-pre-*miR-195* treatment. (**b**–**d**) Quantification of the length, the number and the intersection number of dendrites in hippocampal CA1 pyramidal neurons. (**e**) Typical photomicrographs (top) and tracing images (bottom) of dendritic arborization in cortical pyramidal neurons of 2VO rats with or without lenti-pre-*miR-195* treatment. (**f**–**h**) Quantification of the length, the number and the intersection number of dendrites in cortical pyramidal neurons. (Mean±S.E.M., *n*=20 in hippocampi, *n*=30 in cortices, *P<0.05 *versus* sham; ^#^*P*<0.05 *versus* 2VO. Pri, primary dendrite; Sec, secondary dendrite; Ter, tertiary dendrite; Total, Total dendrite), (**i**) Representative TUNEL photomicrographs in hippocampal CA1 and DG and cortex of 2VO rats with or without lenti-pre-*miR-195* treatment at × 20 magnification. (**j**) Representative Nissl staining photomicrographs in hippocampal CA1 and DG and cortex of 2VO rats with or without lenti-pre-*miR-195* treatment at × 20 magnification

Interestingly, lenti-pre-*miR-195* injection effectively reversed the shortened dendritic length and the lessened dendritic numbers of pyramidal neurons in CA1 (Figures 8a–c) as well as the reduced dendritic crossings by Sholl analysis compared with 2VO rats (Figures 8a and d, *F*_(2, 57)_=29.81, *P*<0.001), while it failed to prevent any changes in granular neurons in the DG region (Supplementary Figures S3a–d). However, lenti-pre-*miR-195* could effectively block the reduction of dendritic length and numbers of pyramidal and granular neurons in the cortex (Figures 8e–g and Supplementary Figures S3e–g) as well as dendritic crossings in both pyramidal (Figure 8h, *F*_(2, 81)_=9.15, *P*<0.001) and granular neurons (Supplementary Figure S3h) of cortical areas relative to 2VO-treated rats.

Furthermore, we observed that lenti-pre-*miR-195* effectively reversed 2VO-induced neuron death in the hippocampal CA1 and cortex as evaluated by TUNEL staining (Figure 8i), and it also increased protein synthesis in neurons, as indicated by increased positive Nissl staining signal compared with that in 2VO-alone rats (Figure 8j). Notably, as assessed by either TUNEL staining or Nissl staining, lenti-pre-*miR-195* did not influence the neuron death and activity in the hippocampal DG area of 2VO rats (Figures 8i and j). The phenomenon was similar to the effect of lenti-pre-*miR-195* on 2VO-triggered dendrite degeneration as evaluated by Golgi impregnation (Supplementary Figures S3a–d).

## Discussion

As a preclinical condition of MCI, CBH was found to be able to induce amyloid-*β* (A*β*) aggregation, tau hyperphosphorylation and cell death.^15, 17, 23, 24, 25^ However, whether CBH could impair dendrite outgrowth, which reportedly precedes cell death and is associated with A*β* toxicity^4, 27^ is unclear. In the present study, we reported that CBH resulted in dendritic remodeling and neuronal death in rat hippocampi and cortices. This process was regulated by the downregulated *miR-195* mediated over-produced N-APP from the cleavage of APP by BACE1 following CBH,^20, 23^ and regulated DR6, which is a necessary and sufficient ligand for N-APP.^5^ In light of our previous studies of *miR-195*-mediated A*β* aggregation,^23^ tau hyperphosphorylation through the activation of Cdk5/p25 (ref. 24) and inactivation of protein phosphatase-2A (PP2A),^25^ our findings here demonstrated that *miR-195* is a key link among the hallmarks of AD and VaD, including A*β* peptide aggregation, tau hyperphosphorylation, synaptic plasticity and neuron death following CBH.

Neuronal networks are the structural basis that enables successful communication between neurons via specific synaptic connections. In these neuronal circuits, a wealth of studies have reinforced the notion that loss of synaptic function, which includes dendritic spine remodeling and axonal degeneration, is a key characteristic of AD.^4, 27, 28, 29^ Recently, the dendritic branching pattern, which dynamically integrates synaptic input by transmitting electrical signals to the soma,^8, 9^ was found to be remodeled, with impaired dendritic ramification, in both Tg2576 mice and sporadic AD rats.^10, 11^ However, whether CBH can induce the remodeling of dendritic morphology is currently unclear. In the present study, we found that CBH by 2VO surgery induced significantly inhibited dendritic branching of the secondary and tertiary dendrites in all pyramidal and granular neurons in the hippocampus and cortex. Interestingly, the primary dendrites were not affected. Axonal degeneration following neuronal insults, such as toxins, metabolic disturbances, infections and mutations growth, occurs in a process called ‘dying-back’ that starts distally and then spreads toward the cell body;^30^ this phenomenon was also found in neurodegenerative diseases including Alzheimer’s and Parkinson’s diseases over weeks or months.^31, 32^ Although there is no report establishing that dendrite degeneration occurs by a dying-back process, the present study provides a clue that chronic brain ischemia may induce a dying-back process of dendrite degeneration. However, more evidence is needed to clarify this process as well as the potential molecular mechanism.

It is known that A*β* aggregation insults synaptic plasticity and impairs dendritic ramification.^4, 10, 11, 27^ Our previous study reported that CBH from 2VO surgery could induce A*β* aggregation by upregulating both APP and BACE1 via low expression of *miR-195*.^23^ Hence, we speculated that *miR-195* may affect the remodeling of dendrites. It has been recognized that the hippocampal subfields have different vulnerability to injury; for example, the pyramidal neurons of the CA1 subfield are most vulnerable to transient ischemia, whereas the neurons of the CA3 subfield and DG are largely resistant.^33, 34, 35^ In the present study, we found that CBH produced similar injury in hippocampal CA1 and DG that was different from transient ischemia.^34^ Interestingly, gain of function of *miR-195* by injection of lenti-pre-*miR-195* directly into the hippocampal CA1 region of 2VO rats significantly reversed the dendritic ramification impairment of pyramidal neurons in the hippocampal CA1 and cortex as well as granular neurons in the cortex; however, it failed to prevent injury to the granular neurons in the DG region of the hippocampus. To clarify this issue, we injected the antisense of *miR-195* (lenti-pre-AMO*-195*) into the CA1 region to mimic the injury of 2VO surgery. We observed that the addition of lenti-pre-AMO*-195* induced the impairment of dendritic ramification of pyramidal neurons in the hippocampal CA1 and cortex as well as granular neurons in the cortex, and these impairments were effectively prevented by co-injection of lenti-pre-*miR-195.* However, it failed to injure the granular neurons in the hippocampal DG region, which was also observed when we evaluated neuron death and cell viability by TUNEL and Nissl staining. The phenomenon may also be explained by differential vulnerability to exogenous intervention or may be because the injected lentiviral vector failed to move to DG from CA1. In addition, the molecular mechanism needs to be clarified further.

Previous studies reported that N-APP, the metabolite of APP, could bind DR6 to trigger axon pruning and neuron death via caspase-6 and caspase-3 respectively.^5^ In addition, A*β* could accelerate the toxic effect of N-APP by upregulating the expression of DR6.^36^ Consistent with a previous study,^6^ we found that CBH induced a significant increase of both N-APP and DR6 accompanied by elevated cleaved caspase-6 and caspase-3 levels. Beyond our prediction, upregulation of *miR-195* by lenti-pre-*miR-195* injection directly to the CA1 region of hippocampi of 2VO rats reversed the elevation of not only N-APP but also DR6 expression. Using three strategies, we found that the *tnfrsf21* gene, which encodes DR6, is the target of *miR-195* and that *miR-195* post-transcriptionally regulates the expression of DR6 by binding with the 3′UTR of DR6 at a position of 1563–1585 bp. Interestingly, masking the action of DR6 using the DR6 antibody and N-APP antibody blocked the action of downregulated *miR-195* in inducing neuron death and increasing cleaved caspase-6 and caspase-3.

Taken together, the results of the present study demonstrate that downregulation of *miR-195* was involved in CBH-induced dendritic remodeling and neuron death by upregulation of APP and BACE1-mediated overproduction of N-APP as well as direct upregulation of DR6 expression simultaneously. In addition, these data provided evidence that upregulation of DR6 could also trigger dendrite remodeling in addition to axon degeneration. However, whether CBH also induces dendritic spine and axonal degeneration needs to be studied further.

## Materials and methods

### Animals

Adult male Sprague Dawley (SD) rats (250–300 g) were purchased from the Animal Center of the Second Affiliated Hospital of Harbin Medical University (Harbin, Heilongjiang Province, China). All animal procedures conformed to the European Parliament Directive (2010/63/EU) and were approved by the Institutional Animal Care and Use Committee at Harbin Medical University (No. HMUIRB-2008-06) and the Institute of Laboratory Animal Science of China (A5655-01).

### Permanent bilateral common carotid artery occlusion (2VO)

2VO rats were prepared according to previous reports.^22, 23^ In brief, after anesthesia, the bilateral common carotid arteries of rats were exposed and permanently ligated with 5–0 silk sutures via a midline cervical incision. The midline incisions were then sutured, and the rats were allowed to recover from anesthesia before being returned to their cages. Sham-operated animals underwent a similar procedure, but without the ligation. Eight weeks after the 2VO surgery, brain tissues were harvested for the subsequent experiments.

### Primary neuron culture

Primary neurons were prepared from postnatal day 0 (P_0_) SD rat pups as previously described.^23^ Neonatal rat cerebral cortices and hippocampi were dissected and dissociated into single cells. The dissociated cells were suspended in DMEM containing 10% fetal bovine serum (FBS, HyClone, Logan, UT, USA) and seeded into a poly-D-lysine coated 6-well plate at a density of 1–2 × 10^6^ cells/well. After 4 h of incubation, the culture medium was replaced with neurobasal medium supplemented with 2% B-27 (Invitrogen, Carlsbad, CA, USA). Subsequently, the neurons were grown in a humidified incubator at 37 °C with 5% CO_2_, and the culture medium was exchanged every 3 days.

### Oligonucleotide synthesis and neuron transfection

*miR-195* mimics, AMO-*miR-195* and NC were synthesized by GenePharma Corporation (Suzhou, China). The DR6-masking antisense ODNs were synthesized by Sangon Biotech Corporation (Shanghai, China). These plasmids were transfected into neurons using X-treme GENE siRNA transfection reagent (catalog #04476093001; Roche, Switzerland) at DIV5 following the manufacturer’s instructions. Forty-eight hours after transfection, they were processed for the subsequent experiments.

### Construction of lentivirus vectors

The detailed method of lentiviral vector construction was the same as in previous reports.^23, 37^ The synthesis and lentivirus packaging of three single-stranded DNA oligonucleotides including pre-*miR195,* pre-AMO-*miR-195* and NC were performed by GeneCopoeia Inc. (Rockville, MD, USA).

### Lentiviral vector injection

After anesthesia, the rats were placed in an animal stereotaxic apparatus. After the skull was exposed, the bilateral hippocampi were located and 2 *μ*l lenti-pre-*miR-195* and/or lenti-pre-AMO-*miR-195* was injected into CA1 of the hippocampus using a 5 *μ*l Hamilton syringe with a 33-gauge tip needle (Hamilton, Bonaduz, Switzerland) at a rate of 30 nl/min following the drilling of a small injection hole. Finally, the skin incision was sutured, and the animal recovered and was returned to its housing. Subsequent experiments were performed 8 weeks after virus injection.^23^

### Dual luciferase reporter assay

HEK293T cells were transfected with *miR-195*, AMO-195 or NC siRNAs as well as psi-CHECK-2-target DNA (firefly luciferase vector) and a blank plasmid using Lipofectamine 2000 transfection reagent (Invitrogen, USA). Luciferase activity was measured with a dual luciferase reporter assay kit (catalog #E1910; Promega, Madison, WI, USA) and luminometer (GloMax 20/20; Promega) after 48 h of transfection. Nucleotide-substitution mutagenesis was performed using direct oligomer synthesis for the 3′UTRs of DR6. All constructs were sequence verified.^23^

### Real-time PCR

Total RNA was purified from the brain tissues using the Trizol reagent and reverse transcribed using TaqMan MicroRNA Reverse Transcription Kit (Applied Biosystems, Carlsbad, CA, USA). Real-time PCR was carried out on a 7900 Fast Realtime System using TaqMan Gene Expression Master Mix (Applied Biosystems). Gene expression was normalized to the internal control.

### Western blot

Protein was extracted from rat brain tissue or primary neurons, subjected to SDS-PAGE, transferred onto nitrocellulose membranes and incubated with primary antibodies against the following: DR6 (1:5000; R&D Systems, Minneapolis, MN, USA), N-APP (1:1000; Millipore, Billerica, MA, USA), and *β*-actin (1:1000; Santa Cruz, Santa Cruz, CA, USA). The membranes were then incubated with fluorescent secondary antibodies (LICOR Biosciences, Lincoln, NE, USA), and the blot bands were captured using an Odyssey Infrared Imaging System (LICOR Biosciences). The signal intensity was analyzed using the Odyssey v. 1.2 software and normalized to the loading control, *β*-actin.

### Immunofluorescence staining

Brain slices at a thickness of 20 *μ*m and primary neurons were fixed with 4% paraformaldehyde, permeabilized and blocked with Triton X-100 and 10% goat serum, and then incubated with the primary antibodies (DR6, Santa Cruz; cleaved caspase-3, cleaved caspase-6, Cell Signaling Technology, Danvers, MA, USA) overnight at 4 °C, followed by secondary antibodies conjugated to Alexa Fluor 488 and Alexa Fluor 594 (Molecular Probes, Eugene, OR, USA) as well as DAPI the next day. Finally, the fluorescence signals were visualized using an LSM 780 laser scanning confocal microscope under the control of LSM software (Olympus FV1000, Japan).

### TUNEL assay

Brain slices at a thickness of 5 *μ*m and primary neurons were rinsed and fixed with 4% paraformaldehyde, and then irreversible DNA damage was analyzed with a fluorescein-based TUNEL kit (Vazyme, Nanjing, China) according to the manufacturer’s protocol. DAPI was used for nuclear staining, and the cells were imaged using the fluorescence microscope. The TUNEL-positive cells per section were then calculated.

### Nissl staining

Brain slices with 20 *μ*m thickness were placed in chloroform for 1 min, then dehydrated with an ethanol gradient. Afterward, they were stained in 0.1% cresyl violet solution for 5 min and subsequently rinsed with distilled water and dehydrated in 95% ethanol. Finally, the slices were cleared in xylene before being mounted with neutral gum. Photographs were obtained at × 20 magnification with a Axio Scope A1 microscope (Carl Zeiss, Germany).

### Golgi staining and sholl analysis

At the 8th week after 2VO surgery or brain injection, the rats were anesthetized and killed by cervical dislocation. The brains were removed and immediately processed using the FD Rapid GolgiStain Kit (FD Neurotechnologies, Columbia, SC, USA) according to the manufacturer’s protocol. Finally, brain slices were observed by brightfield microscopy using a × 20 objective on a Zeiss Axio Scope A1 microscope. An average of 15–30 neurons were randomly examined in each group from 3 brain slices by an investigator blind to the treatment of the rat. The selected neurons were traced and reconstructed using Image-Pro Plus software, and the dendritic complexity was determined by Sholl analysis.

### Statistical analysis

Data are presented as the mean±S.E.M. Statistical analyses were performed using Student’s *t*-test for pairwise comparisons and one-way ANOVA with Tukey *post hoc* tests for comparisons of more than two groups. For Sholl analysis, the effects of group and distance from the soma on dendritic complexity were analyzed using two-factor ANOVA, with repeated measures for distance from the soma, followed by Bonferroni *post hoc* tests. All statistical analyses were performed using SAS 9.1 software (Serial number: 989155; SAS Institute Inc., Cary, NC, USA). Significance was accepted at *P*<0.05, and graphs were generated using GraphPad Prism 5.0 software (La Jolla, CA, USA).
