# Supplementary material for: MicroRNA-195 prevents dendritic degeneration and neuron death in rats following chronic brain hypoperfusion
Source: Cell Death Dis. 2017 Jun 1;8(6):e2850–. doi: 10.1038/cddis.2017.243 (PMC5520902; doi:10.1038/cddis.2017.243)
Supplement: Supplementary Information [file cddis2017243x1.pdf]

MicroRNA-195 prevents dendritic degeneration and neuron death in rats following chronic brain hypoperfusion

Xin Chen[a, d]†, Xue-Mei Jiang[a]†, Lin-Jing Zhao[a]†, Lin-Lin Sun[a],Mei-Ling Yan[a], You Tian[a], Shuai Zhang[a], Ming-Jing Duan[a], Hong-Mei Zhao[a], Wen-Rui Li[a], Yang-Yang Hao[a], Li-Bo Wang[c], Qiao-Jie Xiong[b], Jing Ai[1]*

[a] Department of Pharmacology, (The State-Province Key Laboratories of Biomedicine-Pharmaceutics of China), College of Pharmacy of Harbin Medical University, Harbin, Heilongjiang Province, China, 150086

[b] Department of Neurobiology and Behavior, SUNY at Stony Brook, Stony Brook, NY，USA，1794

[c] Department of medicinal Chemistry and Natural Medicine Chemistry, College of Pharmacy of Harbin Medical University, Harbin, Heilongjiang Province, China, 150086.

[d] Department of Neurosurgery, The First Affiliated Hospital of Harbin Medical University, Harbin, Heilongjiang Province, China, 150001

Running title: miR-195 in neurites degeneration and neuron death.

Corresponding: Jing Ai, Ph. D.,Department of Pharmacology, College of Pharmacy of Harbin Medical University, Harbin Medical University, No.157 Baojian Road, Nangang District, Harbin, Heilongjiang Province, China, 150086; Tel.: +86 451 8667-1354; Fax: +86 451 8667-1354,E-mail address: azhrbmu@126.com or aijing@ems.hrbmu.edn.cn(Jing Ai);

†Xin Chen, Xue-Mei Jiang and Lin-Jing Zhao contributed equally to this work.

Supplementary materials and methods

Animals. Adult male Sprague Dawley (SD) rats used for the experiments were acquired from the Animal Center of the Second Affiliated Hospital of Harbin Medical University (Harbin, Heilongjiang Province, China) with weight ranging from 250-300 g. Animals were housed on a 12 h dark-light artificial cycle (lights on at 7:00 A.M.) at 23±1°C and 55±5% of humidity with food and water available *ad libitum*. Rats used for the operation of permanent, bilateral common carotid artery occlusion (2VO) and stereotaxic injection of the lentiviral vectors were anesthetized by intraperitoneal injection of chloral hydrate (300 mg/kg) and maintained by administrating 0.5-1.0% isoflurane. The depth of anesthesia was monitored by detecting reflexes, heart rate and respiratory rate. Samples for qRT-PCR, Western blot and Golgi staining were obtained from the brains of rats after anesthetized with chloral hydrate (500 mg/kg, intraperitoneal) following by confirmation of death by exsanguination. Frozen brain samples for Immunofluorescence staining, TUNEL and Nissl staining were acquired from the rats after anesthetized with chloral hydrate (300 mg/kg, intraperitoneal) and 0.5-1.0% isoflurane, following by perfusion and fixation with 0.9% saline and 4% paraformaldehyde sequentially. Tissues for primary neuron culturing were from postnatal day 0 (P_0) SD rat pups after administration of 20% isoflurane and confirmation of death by cervical dislocation. All animal procedures were conformed to the European Parliament Directive (2010/63/EU) and were approved by the Institutional Animal Care and Use Committee at Harbin Medical University (No. HMUIRB-2008-06) and the Institute of Laboratory Animal Science of China (A5655-01).

Permanent bilateral common carotid artery occlusion (2VO). 2VO rats were

prepared according to previous reports. In brief, after the rats were anesthetized, the bilateral common carotid arteries (BCCAs) of rats were exposed via a sagittal ventral midline incision, carefully separated from salivatory glands, the respective vagal nerves and accompanying veins without harming these structures. Thereafter, the BCCAs were permanently ligated with 5-0 silk sutures, following by the midline incisions were sutured and lidocaine ointment (AstraZeneca GmbH, Germany) was repeatedly applied to the wounds as needed for analgetic management. Then, rats were transferred to a pre-heated (30°C) recovery box until they had regained complete consciousnes before being returned to their animal cages. Meanwhile, sham operated animals underwent a similar procedure, but without the ligation. Finally, one week after the surgery, brain injection of lentiviral vectors was performed to the 2VO rats, and then, eight weeks after 2VO, brain tissues were harvested for the subsequent experiments.

Primary neuron culture. Primary neuron culture was prepared as previously described. After anesthesia, the rat pups were decapitated, the skins and skulls were removed, and the brains were cut off and placed into a 60mm petri dish filled with cold DMEM (Corning, USA). Then the hippocampi and cortices were dissected carefully from the whole brains after the meninges removed, and were cut into small pieces, which were then dissociated into single cells by 0.125% trypsin (Invitrogen, USA) at 37°C for 15-20 min. Thereafter, the collected cells were suspended in DMEM containing 10% fetal bovine serum (FBS, HyClone, USA) and plated into six-well plates precoated with 10 μg/mL poly-D-lysine (Sigma, USA) at a density of $1\text{-}2\times10^6$ cells/well. After 4 h of incubation, the neuron culture medium was replaced with neurobasal medium (Invitrogen, USA) supplemented with 2% B-27 (Invitrogen,

USA) and 1% antibiotics (Beyotime, China). Subsequently, the neurons were grown in a humidified incubator at 37°C with 5% CO_2. 3 days later, the neurons were treated with 5 μM cytosine arabinoside (Sigma, USA) to inhibit proliferation of dividing cells, and the culture mediums were exchanged every 3 days.

Oligonucleotides synthesis and neuron transfection. *miR-195* mimics (sense: 5'-UAGCAGCACAGAAAUAUUGGC-3'; antisense: 5'-CAAUAUUUCUGUGCUG CUAUU-3') and AMO-*miR-195* (5'-GCCAAUAUUUCUGUGCUGCUA-3') were synthesized by GenePharma Inc. (Suzhou, China). AMO-195 contains 2'-O-methyl modifications at every base and a 3' C3-containing amino linker. Additionally, a scrambled RNA was used as a negative control (sense: 5'-UUCUCCGAACGUGUCACGUAA-3, and antisense: 5'-ACGUGACACGUUCG GAGAAUU-3'). The DR6-masking antisense oligodeoxynucleotides (ODNs) were synthesized by Sangon Biotech Co., Ltd. (Shanghai, China). DR6-masking antisense ODNs was 5'-CCACCAACACAAAACTACACCCGC-3', which masks the binding sites of *miR-195,* located in position 1563-1585 bp of TNFRSF21 3'UTR with deoxynucleotides at both ends of the antisense molecules were locked by a methylene bridge connecting between the 2'-O- and the 4'-C atoms. 75 pmol/ml plasmids above were transfected into neonatal neurons using X-treme GENE siRNA transfection reagent (catalog #04476093001; Roche, Switzerland) at DIV5 following the manufacturer's instructions. The transfection groups were control, NC, *miR-195*, AMO-195, *miR-195* + AMO-195, as well as control, NC, *miR-195*, *miR-195* + DR6-ODN. Forty-eight hours after transfection, neurons were processed for the subsequent experiments.

Construction of lentivirus vectors. The synthesis and lentivirus packaging of pre-*miR195,* pre-AMO-*miR-195* and negative control were performed by GeneCopoeia Inc. (Maryland, USA). In brief, single-stranded DNA oligonucleotides were designed and synthesized with the sequences were as follows: (1) pre-*miR195* ("top strand" oligo: tgctgTAGCAGCACAGAAATATTGGCGTTTTGGCCACTGAC TGACGCCAATATCTGTGCTGCTA) and its complementary chain ("bottom strand" oligo: cctgTAGCAGCACAGATATTGGCGTCAGTCAGTGGCCAAAACGCCAAT ATTTCTGTGCTGCTAc). (2) pre-AMO-*miR-195* ("top strand" oligo: tgctgGCCAATATTTCTGTGCTGCTAGTTTTGGCCACTGACTGACTAGCAGCAG AAATATTGGC) and its complementary sequence ("bottom strand" oligo: cctgGCCAATATTTCTGCTGCTAGTCAGTCAGTGGCCAAAACTAGCAGCACA GAAATATTGGCc). (3) negative control ("top strand" oligo: tgctgAAATGTACTGCGCGTGGAGACGTTTTGGCCACTGACTGACGTCTCCAC GCAGTACATTT) and its complementary sequence ("bottom strand" oligo: cctgAAATGTACTGCGTGGAGACGTCAGTCAGTGGCCAAAACGTCTCCACGC GCAGTACATTTc). We then synthesized the double-stranded oligonucleotides (ds oligo) by annealing the top and bottom strand oligos and cloned these double-stranded DNA oligonucleotides into miRNA expression vector (pcDNA™ 6.2-GW/EmGFPmiR) using the BLOCK-iT pol II miR-RNAi expression vector with the EmGFP kit (Invitrogen, USA). Next, we transformed this miRNA expression vector to the lentivirus expression vector by Gateway recombination technology. Thereafter, the lentiviral particles were obtained by cotransfection of the lentivirus expression vector and packaging plasmids into 293T cells and the viral titer were determined by calculating the appropriate multiplicity of infection (MOI). The titers of the lentivirus used in this experiment were 2.87×10^8 TU/ml for pre-*miR195,* 6.01×10^8 TU/ml for

pre-AMO-*miR-195*. Virus suspensions were stored at -80°C until use and were briefly centrifuged and kept on ice immediately before injection.

Stereotaxic injection of the lentiviral vectors. After anesthesia, the rats were placed onto an animal stereotaxic frame (RWB Life Science, China). After the skull was exposed via a small incision, the bilateral hippocampi were located according to the coordinates relative to the bregma were as follows: AP (anteroposterior), -4.52 mm; ML (mediolateral), ±3.2 mm; DV (dorsoventral), -3.16 mm below the surface of dura using coordinates derived from the atlas of Paxinos and Watson. Then, 2 μL lenti-pre-*miR-195* and/or lenti-pre-AMO-*miR-195* was injected into CA1 of the hippocampus using a 5 μL microsyringe with a 33-gauge tip needle (Hamilton Bonaduz, Switzerland) at a rate of 30 nL/min following the drilling of a small injection hole, the needle was maintained in the place for another 2 min after injection and withdrawn very slowly to prevent the solution backflow. At last, the skin incision was sutured using surgical threads and the animal was recovered on warming pad before returning to its housing cage. Subsequent experiments were performed eight weeks after virus injection [23].

Dual luciferase reporter assay. For the luciferase activity assay, plasmid design and construction was shown as in the below figure: a 1935 bp fragment from the coding region of TNFRSF21 containing the putative binding sequences for *miR-195* (position 1563-1585 of TNFRSF21 CDS) was amplified by PCR and cloned into the psiCHECK-2-control vector.

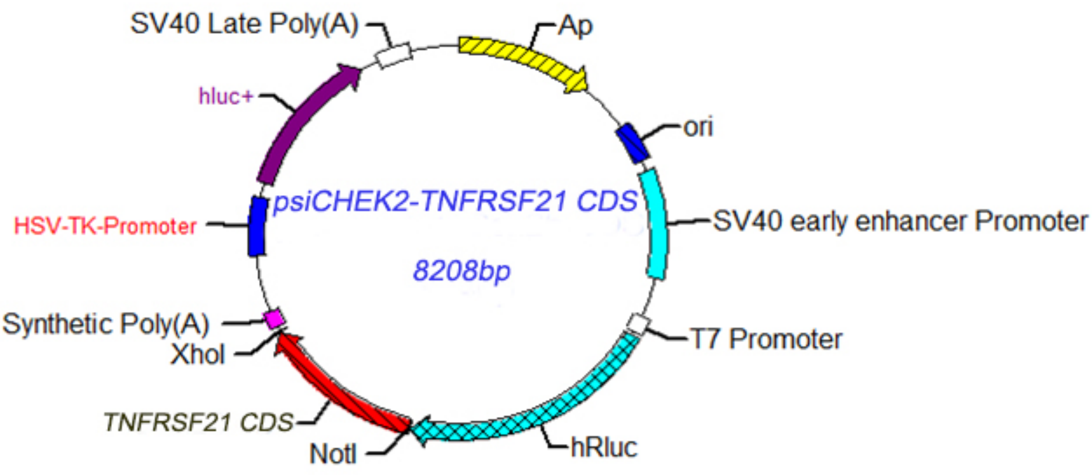

Plasmid design and construction

Mutagenesis nucleotides were carried out using direct oligomer synthesis for the CDS region of TNFRSF21. Point mutations were introduced into a possible *miR-195* binding site located in the coding region of TNFRSF21 (position 1563-1585 bp of TNFRSF21 CDS). MutTNFRSF21 represents that “GTTGGTG” was mutated “ACCAACA” in the position 1579-1585 bp of TNFRSF21 CDS. All constructs were sequence verified. Rat TNFRSF21 CDS and mutTNFRSF21 CDS sequences were shown as following:

TNFRSF21 CDS sequences (blue nucleotide showed the putative chief binding sequences for miR-195):
AACACCGGGGCACTGCGTTCAGGAAATCAGCCTACTGGCAGAGTGATTTCA
TTTCTTTTCTGACTTTTGTGTTTTGGTGTGTGCGTGTATGTATGTGTTTAACA
GAGTATATGGCTGGTGCTTGAGTTTGGGGCTCTTTCTTTCTTTCTTTCTTTCT
TTCCTTCTTTCTTTCTTTTCTCTTCTTGAAAGTGAATGTATAAAGCCTTTTCA
ATGTATAACTGTTCGAAAATGCCCACCACTAAAGTTTTTTTTTTAAGTTCCAT
ATATTCTCCATTTTTGCCTTCTTATATATTTTCAACACTATTCTGTGCACTTTA
AAAACTTAACATAGACGCGGTGTGACTTTTCCCATATGCTGGATTCCAAGA
CTTTGAACTTCTTAAAAAACATAATGGCATCTTGTGACTTCTCTAAGTAGAC
ATAAGTCTGCCAACATCAACGCCTACTTTGTCTGTTTTAATTATCACTGCTCT
CTGTCGTATTGTTTGTCCTTCACGAGCGTTTCTAAGGACGGAGGGAGGCTA
CGACCCTGTTGATGACTGTAACTCTATTAGATTTTGAGTTGTCTTTTTTCATG

TCTTGTTCTATAGTTCATATTCACGACGGAAACTTGACCACACTCTCTATCG
CTCGCTGTATGGTTTTCGTCTGGACATCGTACACTGCTTGTAACTTGTGCTC
CCCTTAATGCTACTAAGCTCTGGGCTGGAGAATGAAATCCTTAAGTCCCCA
GGACTTGCTGTTTCAGTGGCTTGACACCTGGGCCACCAAAGAACTCGATCT
TCATCTTTTAGGAACACCTTTGCTGCACCTTGGAAAACCACTTTATGCCCAG
CTTTGCTTTAAAAGATATTTTTCTTATTTTTTTTATTCCCCTCTCTCTCTCTCT
CTCTCTCTCTCTCTCTCTCTCTCTCTCTCTCTCTCTCTCAATACACATAGTCTATA
GGTCCAGTCTGCCCTCAAGGCCTTGCTGGGTTTCCTTTGTCATCCAACCAC
TTTCGTTAAAAATGGCTGCAGCTGTAAGAACTCCTGTCCGATAAATTTGCA
ACTACGCTCTCTCATTTATCAACCTGTCCTCTGATGCTCAGTTGTCAGACTC
TAATGCAGAGCTAGACGCCCACTGCCTTTGTGTGGGTGGGCTTAGTGGTGA
GGAACTGATATCAGAAAAATGCCTTCAAGTATACTAATTTATTAATAAATATT
AGGTGTTTGTTACTTGCGTGATTTTGGTGTGTTTACTACTTAACTTTCCCCTT
TGCCTCTGTTCAGGTTCTAGCTCTGGTCTTCTCTTGGGGAAGAATGTGTGTT
CTGAAAGCCTCCTCCTGCCCAATCTGTAAATCACACACATTAGCACAAGTC
CCTTTTGGAGAGGTACCCGGAGGTTCAGTGCGCAGGGCTTTGATAAGAACT
GCCAAGCCTTTCCTAGTCTTTCGAGTTAAACCACAGGCTTTTATTTCCTCCA
GGTAATGTTTTTTACCTGTGGGGCAATCTGCACACCTCCACAGACTTCTAGC
TAAAACCTCAGTGTCCCTGACGAACTCCAGAGGGCTTTCTCGGCAGTGAG
GCGGGTGTAGTTTTGTGTTGGTGGGATTTCTCAAGTTCACTGCTGGTTTCCT
TTAGGACCCCGCCTCTGCAGAATTCCAATGACTAGACGCTAGCAATTTTAA
CTTCCTCTTTCTGTGAGAACCTGGAAGTGGATACTGTATGCACTGTGACGC
TCATGTCAGCAACTGGGGAGTGTCTTCTGGTTTCATTCCTCTCCTTGTGGCA
GCTTTCAAGAAGAACACCCCAGTTTCCCTTCCCTCTTGTTGGTGGAAAATA
AGAACTCTGAGGCCAGGTAGTGGTAGTACATGCCTTTAATCTATTGGAAGA
GAGAGGCAGGAGGATATTGAGTTTGAAGCCAGCCTGGGGAGTTCCAGGAC
ATCCATGGCTACCCT

mutTNFRSF21 CDS (red nucleotide represented the mutant sequences):
AACACCGGGGCACTGCGTTCAGGAAATCAGCCTACTGGCAGAGTGATTTCA
TTTCTTTTCTGACTTTTGTGTTTTGGTGTGTGCGTGTATGTATGTGTTTAACA
GAGTATATGGCTGGTGCTTGAGTTTGGGGCTCTTTCTTTCTTTCTTTCTTTCT
TTCCTTCTTTCTTTCTTTTCTCTTCTTGAAAGTGAATGTATAAAGCCTTTTCA
ATGTATAACTGTTCGAAAATGCCCACCACTAAAGTTTTTTTTTTAAGTTCCAT
ATATTCTCCATTTTTGCCTTCTTATATATTTTCAACACTATTCTGTGCACTTTA
AAAACTTAACATAGACGCGGTGTGACTTTTCCCATATGCTGGATTCCAAGA
CTTTGAACTTCTTAAAAAACATAATGGCATCTTGTGACTTCTCTAAGTAGAC
ATAAGTCTGCCAACATCAACGCCTACTTTGTCTGTTTTAATTATCACTGCTCT
CTGTCGTATTGTTTGTCCTTCACGAGCGTTTCTAAGGACGGAGGGAGGCTA
CGACCCTGTTGATGACTGTAACTCTATTAGATTTTGAGTTGTCTTTTTTCATG
TCTTGTTCTATAGTTCATATTCACGACGGAAACTTGACCACACTCTCTATCG
CTCGCTGTATGGTTTTCGTCTGGACATCGTACACTGCTTGTAACTTGTGCTC
CCCTTAATGCTACTAAGCTCTGGGCTGGAGAATGAAATCCTTAAGTCCCCA
GGACTTGCTGTTTCAGTGGCTTGACACCTGGGCCACCAAAGAACTCGATCT
TCATCTTTTAGGAACACCTTTGCTGCACCTTGGAAAACCACTTTATGCCCAG

CTTTGCTTTAAAAGATATTTTTCTTATTTTTTTATTCCCCTCTCTCTCTCTCT
CTCTCTCTCTCTCTCTCTCTCTCTCTCTCTCTCTCTCAATACACATAGTCTATA
GGTCCAGTCTGCCCTCAAGGCCTTGCTGGGTTTCCTTTGTCATCCAACCAC
TTTCGTTAAAAATGGCTGCAGCTGTAAGAACTCCTGTCCGATAAATTTGCA
ACTACGCTCTCTCATTTATCAACCTGTCCTCTGATGCTCAGTTGTCAGACTC
TAATGCAGAGCTAGACGCCCACTGCCTTTGTGTGGGTGGGCTTAGTGGTGA
GGAACTGATATCAGAAAAATGCCTTCAAGTATACTAATTTATTAATAAATATT
AGGTGTTTGTTACTTGCGTGATTTTGGTGTGTTTACTACTTAACTTTCCCCTT
TGCCTCTGTTCAGGTTCTAGCTCTGGTCTTCTCTTGGGGAAGAATGTGTGTT
CTGAAAGCCTCCTCCTGCCCAATCTGTAAATCACACACATTAGCACAAGTC
CCTTTTGGAGAGGTACCCGGAGGTTCAGTGCGCAGGGCTTTGATAAGAACT
GCCAAGCCTTTCCTAGTCTTTCGAGTTAAACCACAGGCTTTTATTTCCTCCA
GGTAATGTTTTTTACCTGTGGGGCAATCTGCACACCTCCACAGACTTCTAGC
TAAAACCTCAGTGTCCCTGACGAACTCCAGAGGGCTTTCTCGGCAGTGAG
GCGGGTGTAGTTTTGTACCAACAGGATTTCTCAAGTTCACTGCTGGTTTCCT
TTAGGACCCCGCCTCTGCAGAATTCCAATGACTAGACGCTAGCAATTTTAA
CTTCCTCTTTCTGTGAGAACCTGGAAGTGGATACTGTATGCACTGTGACGC
TCATGTCAGCAACTGGGGAGTGTCTTCTGGTTTCATTCCTCTCCTTGTGGCA
GCTTTCAAGAAGAACACCCCAGTTTCCCTTCCCTCTTGTTGGTGGAAAATA
AGAACTCTGAGGCCAGGTAGTGGTAGTACATGCCTTTAATCTATTGGAAGA
GAGAGGCAGGAGGATATTGAGTTTGAAGCCAGCCTGGGGAGTTCCAGGAC
ATCCATGGCTACCCT

The sequence of *miR-195* mimic is 5’-UAGCAGCACAGAAAUAUUGGC-3’ synthesized based on the sequence of rno-*miR-195* (miRBase Accession No. MI0000939); that of miR-NC is 5’-UUCUCCGAACGUGUCACGUAA-3’; the sequence of the antisense 2’-O-methyl (2’-O-Me) oligonucleotide for *miR-195* (AMO-*miR-195*) is 5’-GCCAAUAUUUCUGUGCUGCUA-3’, that of inhibitor-NC is 5’-UUCUCCGAACGUGUCACGUTT-3’. HEK293T cells (plated at 40%~50% confluence) were transfected with 20 μmol/l *miR-195*, AMO-*miR-195*, or NC siRNAs as well as 0.5 μg of psi-CHECK^{TM}-2-target DNA (firefly luciferase vector) and 1 μl blank plasmid using Lipofectamine 2000 transfection reagent (Invitrogen, USA) according to the manufacturer’s instructions. After 48 h transfection, firefly and renilla luciferase activities, as indicated by relative luminescence units (RLU) were determined using luciferase assay kits (catalog #E1910; Promega, USA) and

luminometer (GloMax^{TM} 20/20; Promega, USA) according to the manufacturer's instructions.

Quantitative real-time PCR (qRT-PCR). Total RNA was purified from the brain tissues or primary neurons using the Trizol Reagent (Invitrogen, USA), then they were reverse transcribed using TaqMan® MicroRNA Reverse Transcription Kit (catalog #4366596, Applied Biosystems, USA). Thereafter, real time PCR was carried out on a 7900 Fast Realtime System (Applied Biosystems, USA) using TaqMan® Gene Expression Master Mix (catalog #1108123, Applied Biosystems, USA) with the protocol was as following: (1) 95ºC, 10min; (2) 95ºC, 15s; (3) 60ºC, 1min (repeat (2) and (3) for 40 cycles), the primers were as follows: *miR-195* forward: 5'-GGGGTAGCAGCACAGAAAT -3' and m*iR-195* reverse: 5'-TCCAGTGCGTGTCGTGGA -3; *U6* forward: 5'-GCTTCGGCAGCACATATACTAAAAT -3' and *U6* reverse: '-CGCTTCACGAATTTGCGTGTCAT -3'. All reactions were performed in triplicate, and the results were normalized against the internal control (*U6*) using the δ–δ CT method.

Western blot. Both the total protein samples were extracted from hippocampus and cortex of rats or primary cultured neurons for immunoblotting analysis. For the tissue protein extraction, frozen tissue was homogenized with 100 μl lysis buffer contained 40% SDS, 60% RIPA and 1% protease inhibitor (Roche, Switzerland) in each 20 mg brain tissue. For the cell protein analysis, cultured neurons were homogenized with 30-40 μl lysis buffer contained 99% RIPA and 1% protease inhibitor in each well of 6-well plate. The homogenates were then centrifuged at 13,500 rpm for 30 min at 4°C

and the clarified supernatants (containing cytosolic and membrane fractions) were carefully removed. The concentrations of extracted total protein were then measured spectrophotometrically using a BCA kit (Beyotime, China). Next, the protein samples were prepared by adding protein loading buffer according to their concentrations and were denatured in a dry bath at 100°C for 5 min. Thereafter, Protein were fractionated by 12.5% SDS-PAGE gel (Epizyme, China) using Tricine SDS running buffer, according to the manufacturer's instructions and were transferred onto nitrocellulose membranes using Tris-Glycine transfer buffer (20% methanol). The membranes were then blocked with 5% nonfat milk (BD, USA) in TBS-T buffer and incubated with the primary antibodies at 4°C overnight. The primary anti-DR6 antibody (Cat# AF144; 1:5000, R&D Systems, USA), anti- N-APP antibody (Cat# MAB348; 1:1000; Millipore, USA) were used and β-actin (Cat# sc-47778; 1:1000, Santa Cruz, USA) was selected as an internal control. Next day, the membranes were subsequently incubated with fluorescence secondary antibodies (LICOR Biosciences, USA) at room temperature for 1 hour. The blot bands were then captured using Odyssey Infrared Imaging System (LICOR Biosciences, USA) and quantified with Odyssey v1.2 software by measuring the band intensity (area × OD) in each group and normalizing to the internal control.

Immunofluorescence staining. For tissue slices preparation, the rats were deeply anaesthetized and perfused transcardially using 50 mL of PBS (pH 7.4), followed by 250 mL of 4% paraformaldehyde at 8th weeks after 2VO surgery and brain injection. Thereafter, the rats were decapitated and the brains were fixed in 4% paraformaldehyde at 4°C for 24 h, followed by dehydration with gradient sucrose solution. The brains were subsequently frozen and sectioned coronally using a

cryostat microtome (Leica, Germany) at a thickness of 20 μm, and then the slices were mounted on glass slides. For cell slices preparation, the neonatal rat neurons were cultured on plate covered by glass coverslips. Thereafter, brain slices were incubated in citrate buffer at 95°C for 15 min to retrieve antigen and were fixed with 4% paraformaldehyde in PBS for 30 min, followed by permeabilization and blocking with 0.3% Triton X-100 and 10% goat serum albumin in PBS for 60 min, while, the neurons were fixed with 4% paraformaldehyde in PBS for 15 min, followed by permeabilization and blocking with 0.1% Triton X-100 and 10% goat serum albumin in PBS for 60 min at room temperature. After blocking, neurons or brain sections were incubated with the primary antibodies overnight at 4°C, and then were washed and incubated with the appropriate fluorescent secondary antibodies conjugated to Alexa Fluor 488 or Alexa Fluor 594 (Molecular Probes, USA) for 1h as well as DAPI for 15 min at room temperature the next day. The primary anti-DR6 antibody (Cat# sc-25772; 1:50, Santa Cruz, USA), anti-cleaved caspase-3 antibody (Cat# 9661; 1:300, Cell Signaling Technology, USA) and anti-cleaved caspase-6 antibody (Cat# 9761; 1:300, Cell Signaling Technology, USA) were used for tissue staining, while, the primary anti-DR6 antibody (Cat# sc-25772; 1:50, Santa Cruz, USA), anti-cleaved caspase-3 antibody (Cat# 9661; 1:500, Cell Signaling Technology, USA) and anti-cleaved caspase-6 antibody (Cat# 9761; 1:500, Cell Signaling Technology, USA) were used for cell staining. Finally, the slides were mounted with mounting mediums and the fluorescence signals were visualized using an LSM 780 laser-scanning confocal microscope under the control of LSM software (Olympus FV1000, Japan) at the 20× objective.

TUNEL assay. For cells, neurons were cultured on glass coverslips in 6 well plates

prior to treatment. For tissues, the frozen rat brains were obtained as previous description in methods of Immunofluorescence staining and were sectioned coronally using a cryostat microtome (Leica, Germany) at a thickness of 5 μm. Thereafter, they were rinsed and fixed with 4% paraformaldehyde, and then irreversible DNA damage was analyzed by fluorescein-based TUNEL kit (Vazyme, China) based on TdT-mediated dUTP nick end labeling (TUNEL) according to the manufacturer's protocol. In brief, the cell and tissue slides were penetrated with Proteinase K (20 μg/ml) for 5-10 min at room temperature. After washing in PBS and equilibrating with Equilibration Buffer, they were incubated in TUNEL reaction mixture including Bright Green Labeling Mix and Recombinant TdT Enzyme for 60 min in moisture condition at 37°C. Then, they were rinsed with PBS and were stained with DAPI for nuclear labeling. Finally, the slides in each group from 3 brains were captured randomly using the fluorescence Axio Scope A1 microscope (Carl Zeiss, Germany) at the magnification of 20× and the TUNEL-positive cells per section were then calculated. To minimize the introduction of bias into the cell imaging and counting studies, the experimenter was blinded to the group being analyzed.

Nissl staining. The frozen rat brains were obtained as previous description in methods of Immunofluorescence staining and were sectioned coronally using a cryostat microtome (Leica, Germany) at a thickness of 20 μm. Thereafter, the brain slices were placed in chloroform for 1 min, then they were dehydrated according to the following sequence: 70% (v/v) ethanol, 95% (v/v) ethanol, and 100% (v/v) ethanol, each dip carried out for 1 min. The slices were subsequently transferred to distilled water for a while and placed in 0.1% cresyl violet solution (Beyotime, China) for 5 min at room temperature. Following this, the slices were rinsed in distilled water for a second, then

were dehydrated in 95% ethanol for 2 min, and subsequently were cleared in xylene for 5 min. Finally, the slices were overlayed with coverslips in neutral gum mounting medium. The Photographs including tissue structures and the morphology of neurons in the cerebral cortex and hippocampus were obtained by Axio Scope A1 microscope (Carl Zeiss, Germany) at the 20× objective. The resulting Nissl staining displayed dark blue color in a normal region, but light blue color in the infarct area of the brain.

Golgi staining and Sholl analysis. At the 8th weeks after 2VO surgery or brain injection, rats were anesthetized deeply and sacrificed. The brains were removed and immediately processed using the FD Rapid GolgiStain Kit (FD Neurotechnologies, USA) according to the manufacturer's protocol. Briefly, after removal of the brain, the brain was rinsed with double distilled water and placed immediately into Golgi impregnation solution (1: 1 mixture of FD Solution A: B) in an opaque container. The impregnation solution was changed the following day and brains were stored in the dark for 14 days at room temperature. Brain tissue was then transferred to FD Solution C. After 24 hours, the solution was replaced with fresh solution C, and the brain was stored at 4°C for 2 days. Thereafter, brains were sectioned on 100 μm thickness at −22°C using cryostat microtome (Leica, Germany), the slices were mounted on gelatin coated slides and air dried at room temperature in the dark for at least 4 hours (or overnight). The slides were then stained with FD Solution D and E, dehydrated with gradient ethanol, cleared in xylene and coverslipped with neutral gum mounting medium according to the manufacturer's instruction. Finally, brain slices were observed under brightfield using Axio Scope A1 microscope (Carl Zeiss, Germany) at the magnification of 20×. The neuron inclusion criteria for analysis were that neurons containing full Golgi-cox impregnation without breaks along the

dendrites, and no obstructions by neighboring cells. To ensure that only complete cells were reconstructed, the selected cells were located close to the center of the section, and superficial cells, with significant breaks at the surface of the section, were excluded from analysis. For this study, an average of 15-30 neurons was randomly examined in each group from 3 brains by an investigator blind to the treatment of the rat. The selected neurons were traced, and reconstructed using Image-Pro Plus software. Finally, the length and number of total, primary, secondary and tertiary dendrite of pyramidal and granular neurons in hippocampal and cortex of rats were measured, meanwhile, the dendritic complexity was determined by Sholl analysis.

Statistical analysis. Data are presented as the mean ± SEM. Statistical analyses were performed using Student's t-test for pairwise comparison and one-way ANOVA with Tukey post hoc tests for comparisons of more than two groups. For Sholl analysis, the effects of group and distance from the soma on dendritic complexity were analyzed using two-factor ANOVA, with repeated measures for distance from the soma, followed by Bonferroni post hoc tests. All statistical analyses were performed using SAS 9.1 software (Serial number: 989155; Institute Inc.), two-tailed P values < 0.05 was considered statistically significant, and graphs were generated using GraphPad Prism 5.0 software.

Supplementary Figures

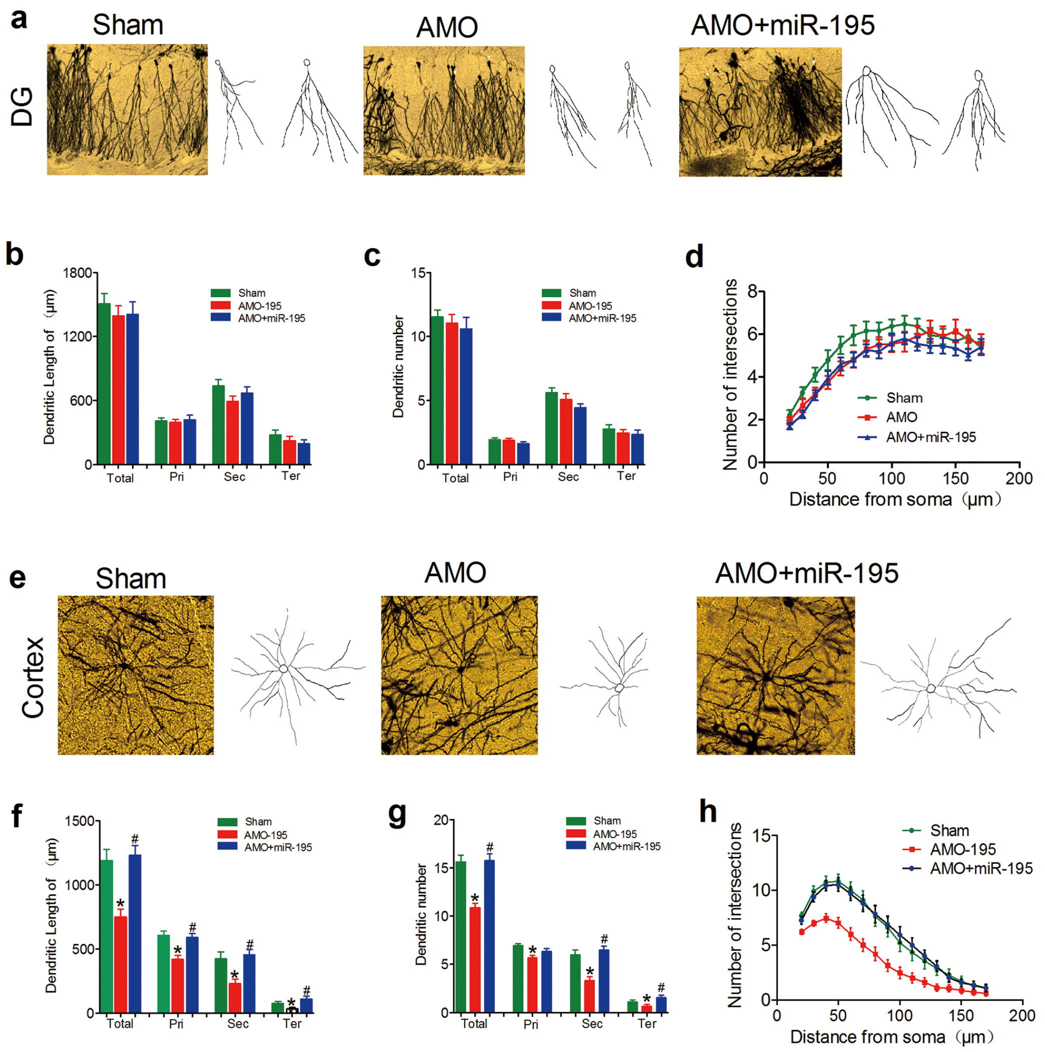

Figure S1 Effects of lenti-pre-AMO-*miR-195* on dendritic complexity deficits of granular neurons in hippocampus and cortex. (**a**) Typical photomicrographs (left) and single tracing images (right) of dendritic arborization in hippocampal DG granular neurons from rats treated with lenti-pre-AMO-*miR-195*, lenti-pre-AMO-*miR-195* + lenti-pre-*miR-195*, and NC. (**b-d**) Quantification of the length, the number of total, primary, secondary, tertiary dendrites, and the number of intersections of dendrites with concentric circles distanced from the soma in hippocampal DG granular neurons from rats treated with lenti-pre-AMO-*miR-195*, lenti-pre-AMO-*miR-195* + lenti-pre-*miR-195*, and NC. (**e**) Typical photomicrographs (left) and single tracing

images (right) of dendritic arborization in cortex granular neurons from rats treated with lenti-pre-AMO-*miR-195*, lenti-pre-AMO-*miR-195* + lenti-pre-*miR-195*, and NC. (**f-h**) Quantification of the length, the number of total, primary, secondary, tertiary dendrites, and the number of intersections of dendrites with concentric circles distanced from the soma in cortex granular neurons from rats treated with lenti-pre-AMO-*miR-195*, lenti-pre-AMO-*miR-195* + lenti-pre-*miR-195*, and NC. (Data are reported as mean ± SEM, n = 20 cells from three rats for each group in hippocampus, n = 30 cells from three rats for each group in cortex. **P* < 0.05 versus Sham; #*P* < 0.05 versus lenti-pre-AMO-*miR-195*. Abbreviations: Total, total dendrite; Pri, primary dendrite; Sec, secondary dendrite; Ter, tertiary dendrite).

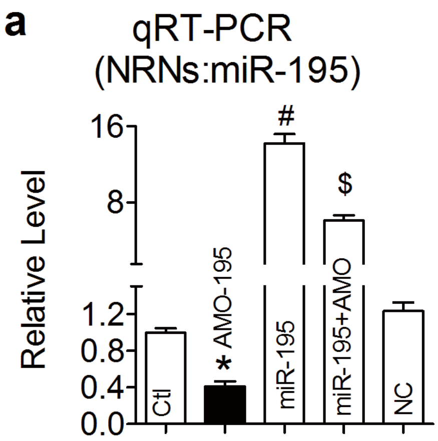

Figure S2 The expression level of *miR-195* in neurons after transfection with *miR-195* or/and AMO-195 by qRT-PCR. 48 h transfection of *miR-195* or AMO-195 could significantly upregulate or downregulate *miR-195* expression levels in NRNs (n = 6 for each group, **P* < 0.05 versus Ctl, #*P* < 0.05 versus AMO-195, $*P* < 0.05 versus *miR-195*).

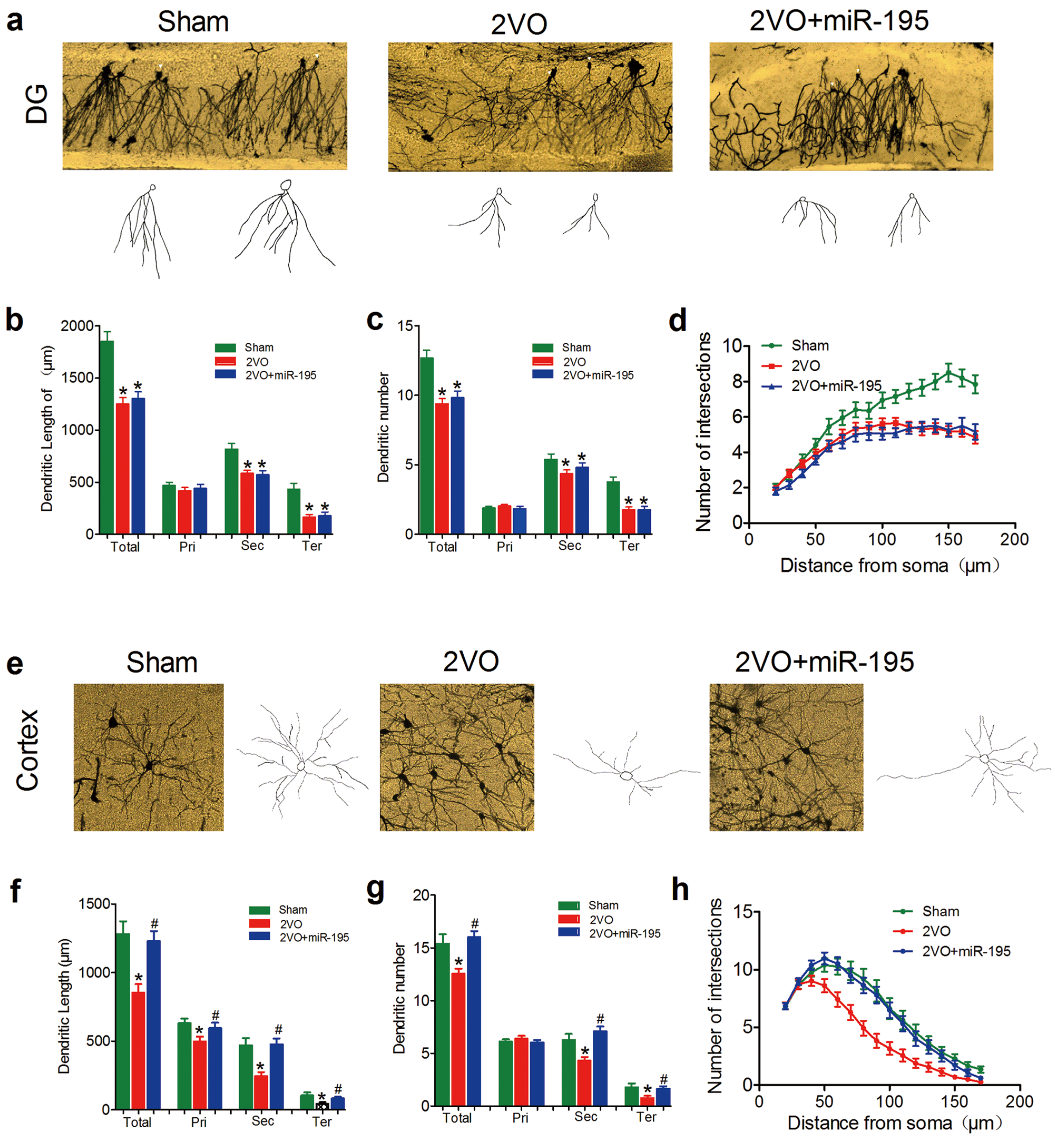

Figure S3 The effect of *miR-195* supplement on dendritic complexity deficits in granular neurons in 2VO rats. (**a**) Typical photomicrographs (top) and single tracing images (bottom, arrows) of dendritic arborization in hippocampal DG granular neurons of 2VO rats with or without lenti-pre-*miR-195* treatment. (**b-d**) Quantification of the length, the number of total, primary, secondary, tertiary dendrites, and the number of intersections of dendrites with concentric circles distanced from the soma in hippocampal DG granular neurons. (**e**) Typical photomicrographs (left) and single tracing images (right) of dendritic arborization in

cortex granular neurons of 2VO rats with or without lenti-pre-*miR-195* treatment. (**f**-**h**) Quantification of the length, the number of total, primary, secondary, tertiary dendrites, and the number of intersections of dendrites with concentric circles distanced from the soma in cortex granular neurons. (Data are reported as mean ± SEM, n = 20 cells from three rats for each group in hippocampus, n = 30 cells from three rats for each group in cortex, **P* < 0.05 versus sham; #*P* < 0.05 versus 2VO. Abbreviations: Total, total dendrite; Pri, primary dendrite; Sec, secondary dendrite; Ter, tertiary dendrite).
